# 

**Published:** 1877-06

**Authors:** 


*CONTENTS.*

| | Page |
|---|---|
| Checked Perspiration | 151 |
| Remedy for a Cough | 153 |
| Throat Ail | 156 |
| Influences | 158 |
| Agriculture | 159 |
| Punishing Children | 163 |
| Clerical Recreations | 164 |
| Care of the Feet | 166 |
| Popular Fallacies | 166 |
| Exercise | 169 |
| Health of Children | 171 |
| The Works of Dr. Hall | ~~175~~ 173 |
| Reason in All Things | ~~175~~ 173 |
| Valuable Remedies | ~~176~~ 174 |
| Bartholdi's Colossal "Liberty" | ~~177~~ 175 |
| Life Insurance | ~~178~~ 176 |

